# Efficacy and safety of chloroquine and hydroxychloroquine in the treatment of patients with COVID-19 combined with diabetes mellitus: A protocol for systematic review and meta-analysis

**DOI:** 10.1097/MD.0000000000022031

**Published:** 2020-09-11

**Authors:** Yan Liu, Xiaoxu Fu, Chunguang Xie

**Affiliations:** a Hospital of Chengdu University of Traditional Chinese Medicine; b Chengdu University of Traditional Chinese Medicine, Chengdu, Sichuan Province, China.

**Keywords:** chloroquine, COVID-19, diabetes mellitus, hydroxychloroquine, meta-analysis, protocol, systematic review

## Abstract

**Background:**

Diabetes is a common chronic metabolic disease. COVID-19 is a large-scale infectious disease that broke out in 2019, and 212 countries have now been infected with this infectious disease. Some studies have shown that COVID-19 combined with diabetes is an independent risk factor for death or other adverse outcomes. There is currently no specific and effective drug treatment. More and more people have realized that the low-cost CQ and its derivative HCQ have antiviral and anti-inflammatory capabilities and may play a huge role in the fight against COVID-19. At the same time, HCQ can be used as an oral hypoglycemic agent and has the effect of lowering blood glucose. However, there is no evidence-based medicine to confirm the effectiveness and safety of CQ and HCQ in the treatment of COVID-19 patients with diabetes. Therefore, we will conduct a systematic review and meta-analysis to synthesize the existing clinical evidences.

**Methods and analysis:**

Chinese literature comes from CNKI, Wanfang, VIP, CBM databases. English literature mainly searches Cochrane Library, PubMed, Web of Science, EMBASE. We will retrieve each database from December 2019 to August 2020. At the same time, we will look for clinical trial registration and gray literature. This study only included clinical randomized controlled trials. The reviewers independently conduct literature selection, data analysis, quality analysis, and evaluation. The primary outcomes include Sputum virus nucleic acid negative time, lung imaging improvement time, mortality rate, mechanical ventilation rate, ICU hospitalization time, hospitalization time, clinical improvement, symptoms Improvement, fasting blood glucose, 2-hour postprandial blood glucose, glycosylated hemoglobin, fasting insulin, adverse reactions, etc. Finally, we will conducted a meta-analysis through Review Manager Software version 5.3.

**Results:**

The results will be published in peer-reviewed journals and presented at a relevant conference.

**Conclusion:**

This study will explore the effectiveness and safety of CQ and HCQ in the treatment of COVID-19 patients with diabetes. It will provide evidence-based medical evidence for CQ and HCQ in the treatment of diabetes with COVID-19.

**Registration number:**

INPLASY202070109.

## 1 Introduction

Coronavirus disease 2019 (COVID-19) is a new infectious disease^[1]^ that has caused 10,420,325 infections and 508,467 deaths worldwide. Diabetes mellitus (DM) is a chronic metabolic disease with high blood glucose as the main manifestation, accompanied by multi-system and multi-organ damage.^[2]^ Many studies have shown that diabetes accounts for 10.1% to 20.0% of COVID-19 patients, and 22.2% of critically ill patients.^[3–6]^ It is not clear whether diabetic patients are more susceptible to COVID-19, nor is it clear whether diabetic patients are more prone to hyperglycemia after being infected with COVID-19, but there are many reports on the relationship between COVID-19 and diabetes. It is speculated that angiotensin converting enzyme 2 (ACE2) may give a reasonable explanation for this.^[7–8]^ At the same time, hyperglycemia may be a risk factor for serious infections, and might be an independent risk factor for COVID-19 from mild to severe.^[9]^ Therefore, patients with diabetes and COVID-19 may need special attention and clinical care.

In the current global shortage of medical resources and lack of effective drugs, the low-cost antimalarial drugs chloroquine (CQ) and its derivative hydroxychloroquine (HCQ) have attracted more and more attention.^[10]^ CQ has been proven to have a wide range of antiviral effects.^[11]^ According to published studies, CQ may interfere with the growth and reproduction of the virus by affecting the ACE2 receptor on the surface of the host cell, leading to glycosylation defects.^[12–15]^ On the other hand, CQ is weakly alkaline and can interfere with the virus by increasing the pH of the body.^[13]^ HCQ and CQ have exactly the same structure and mechanism of action, except for an additional hydroxy moiety in 1 terminal in HCQ.^[16]^ Both of them can change the pH of intracellular organelles. Both the agents are considered to be effective tools against Severe Acute Respiratory Syndrome Coronavirus 2 (SARS-CoV-2). Many studies have shown that HCQ inhibits SARS-CoV-2 in vitro, and its concentration can be reached in human lung tissue.^[17]^ At the same time, HCQ, as a third-or fourth-line oral hypoglycemic agent for the treatment of type 2 diabetes, has a significant effect on lowering glycosylated hemoglobin. Therefore, the researches of HCQ and CQ have exciting potential for diabetic patients infected with COVID-19.

Therefore, this article aims to explore the effectiveness and safety of CQ and HCQ in the treatment of COVID-19 patients with diabetes. This result may provide a new basis for the clinical treatment of COVID-19 with diabetes.

## 2 Methods and analysis

### 2.1 Study registration

We have completed the registration of the systematic review protocol on the INPLASY website as INPLASY202070109 (https://inplasy.com/inplasy-2020-7-0109/). It is reported on the basis of Cochrane Handbook for Systematic Reviews of Interventions, and the Preferred Reporting Items for Systematic Reviews and Meta-analysis Protocol (PRISMA),^[18]^ and the important protocol revisions will be recorded in the full review.

### 2.2 Inclusion and exclusion criteria

#### 2.2.1 Study design

Our research will be limited to randomized controled trials (RCT). Meanwhile, repeated publications of the same study, reviews, letters, abstracts, or animal experiments are excluded.

#### 2.2.2 Participants

All patients who were diagnosed as COVID-19 will be included in our research, there will be no limitation about age, region, gender, disease severity, and other factors.

#### 2.2.3 Interventions and comparators

The experimental group is patients diagnosed with COVID-19 and diabetes, while the control group is COVID-19 patients without diabetes. Both groups of patients received conventional COVID-19 treatment. The experimental group received conventional diabetes treatment recommended by the American Diabetes Association (ADA) guidelines,^[19]^ including diet, exercise, hypoglycemia and lipid-lowering treatment and chloroquine/hydroxychloroquine treatment, and the control group received placebo or no treatment.

#### 2.2.4 Outcomes

The primary outcomes include Sputum virus nucleic acid negative time, lung imaging improvement time, mortality rate, mechanical ventilation rate, intensive care unit (ICU) hospitalization time, hospitalization time, clinical improvement, symptoms Improvement, fasting blood glucose, 2-hour postprandial blood glucose, glycosylated hemoglobin, fasting insulin, adverse reactions, etc.

### 2.3 Study search

We use a combination of title words and free words as a search strategy, which is jointly decided by the reviewers. Electronic databases include CNKI, Wanfang, VIP, CBM database, Cochrane Library, PubMed, Web of Science, EMBASE, etc. At the same time, we will search for clinical trial registration and grey literature in Clinicaltrials.gov, the World Health Organization International Clinical Trials Registry Platform and China Conference Paper Database to make up for the lack of electronic databases. We will search each database from December 2019 to August 2020. The language of the publications will be limited to English and Chinese. We will give a detailed search process in Table 1. Adjust different search methods in the light of different Chinese and English databases.

**Table 1:**
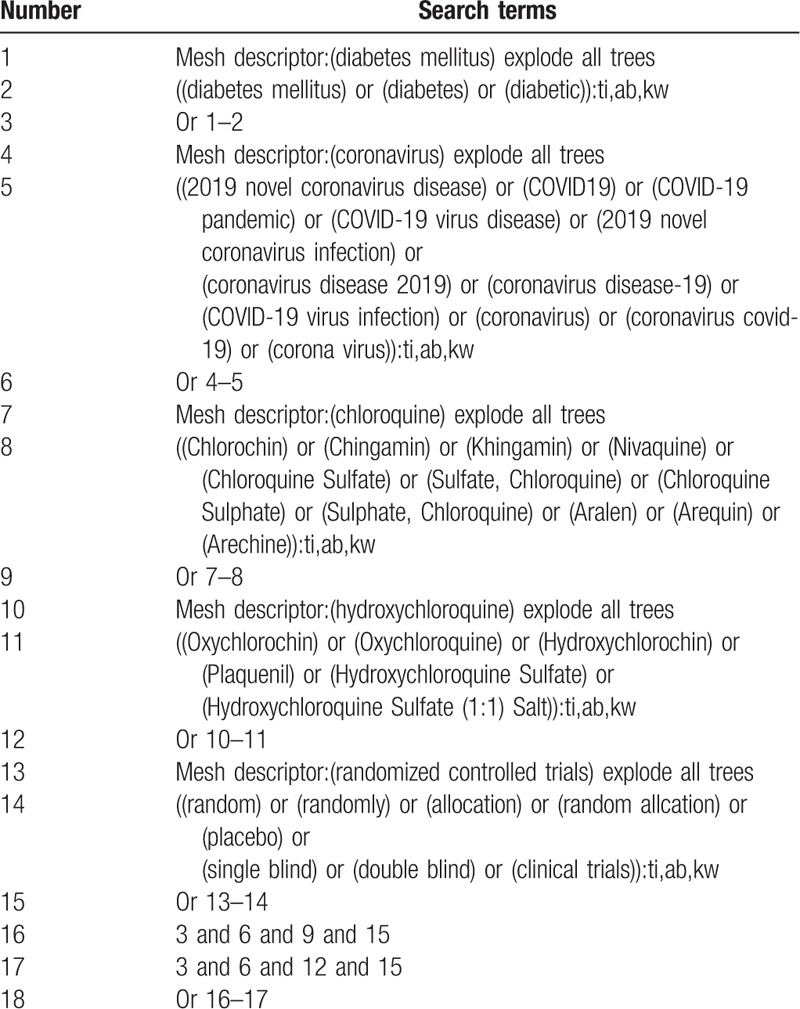
Example of Cochrane search strategy.

### 2.4 Data collection and analysis

#### 2.4.1 Selection of studies

**Figure 1:**
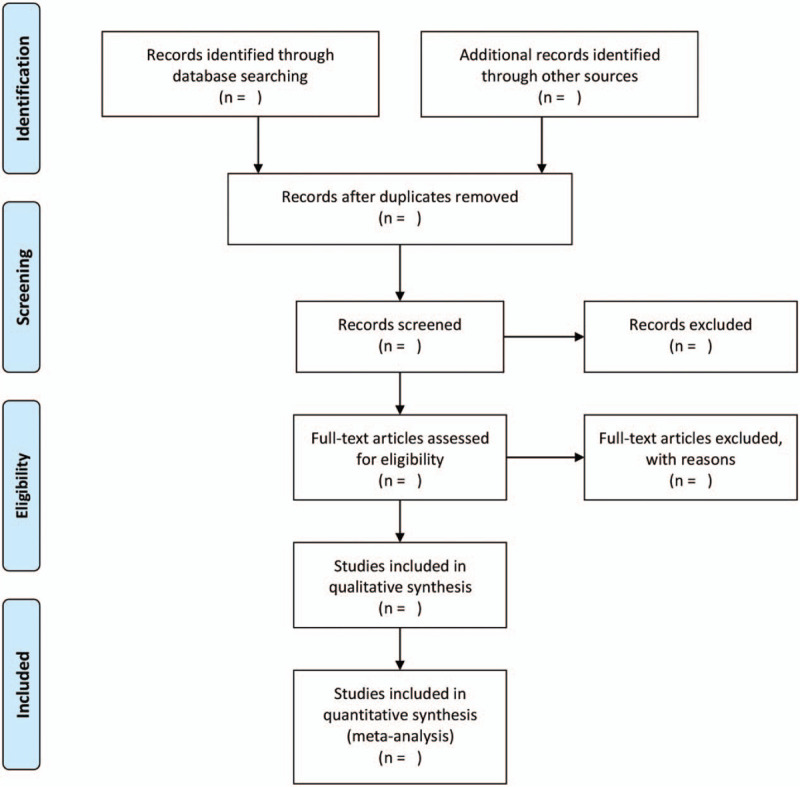
Flow chart of study selection.

Import all the required literature into the endnote x^9^ software. Two independent reviewers screened the documents that did not meet the inclusion criteria of this study by reading the abstract and title, and then read the full text to decide whether to include them. In case of disagreement in the above process, this agreement will be negotiated with a third party. In addition, we will use a flowchart (Fig. 1) to show the process of exclusion causes and study selection.

#### 2.4.2 Data extraction and management

Qualified literature data will be extracted into Microsoft Excel by 2 reviewers. We will extract the following information: title, author, year, sample size, age, gender, course of disease, intervention measures, outcomes, and adverse reactions. If the reported data is insufficient or ambiguous, we will contact the corresponding author for complete information. If we are unable to get in touch with the author, we will exclude the study because of missing important information.

### 2.5 Risk of bias assessment

All the included studies will be evaluated according to the guidelines of Cochrane Handbook for Systematic Reviews of Interventions. The evaluation items are as follow: random sequence generation, allocation concealment, blinding participants and personnel, blinding evaluation of results, incomplete outcome data, selective result reporting, and other biases. The quality of each trial is categorized into “low”, “high”, or “unclear” risk of bias.^[20]^ When there are different opinions, the 2 reviewers can discuss or seek third-party consultation to reach a consistent conclusion.

### 2.6 Data analysis

We will use Review Manager software version 5.3 provided by Cochrane Collaboration to analyze the data. 95% RR was used to represent the dichotomous data. Continuous data will be represented by MD or SMD. When *I*^2^ < 50%, *P* > .01 indicates that the study has no statistical heterogeneity, a fixed-effect model will be used; on the contrary, when *I*^2^ ≥ 50%, *P* < .01, indicating the existence of considerable heterogeneity, the random effects model will be used for analysis.^[21]^ In addition, based on the different causes of heterogeneity, we will further conduct subgroup or sensitivity analysis. If meta-analysis cannot be performed, we will conduct a descriptive analysis instead.

### 2.7 Subgroup analysis

We will divide patients diagnosed with COVID-19 into experimental group and control group according to whether they have diabetes, and then conduct subgroup analysis based on different reasons such as age, gender, different forms of intervention, treatment process, drug dosage, etc.

### 2.8 Sensitivity analysis

To evaluate the robustness of the primary outcome measures, we will eliminate the low-quality studies and combine the data to assess the impact of the sample size, study quality, statistical methods, and missing data on the meta-analysis results.

### 2.9 Publication bias assessment

If there are more than 10 studies in the meta-analysis, we will evaluate the symmetry of the funnel plot to examine the publication bias and interpret the results carefully.^[22–23]^

### 2.10 Grading the quality of evidence

The quality of the evidence for the entire study will be assessed through the “grades of recommendations assessment, development, and evaluation (GRADE)” standard established by the WHO and international organizations. For more clarity, the GRADE system divides the quality of evidence into: “high”, “medium”,“low”, and “very low”. The GRADE profiler 3.2 will be employed for analysis.

### 2.11 Patient and public involvement

Patients and the public will not be involved in this study.

### 2.12 Ethics and dissemination

Since our research is a protocol for systematic review and meta-analysis, ethical approval is not required. Our research results will also be published in peer-reviewed journals and presented at a relevant conference.

## 3 Discussion

Diabetes is a common chronic metabolic disease. COVID-19 is a large-scale infectious disease that broke out in 2019, and 212 countries have now been infected with this infectious disease. Some studies have shown that COVID-19 combined with diabetes is an independent risk factor for death or other adverse outcomes. There is currently no specific and effective drug treatment. More and more people realize that the low-cost CQ and its derivative HCQ have antiviral and anti-inflammatory capabilities and may play a huge role in the fight against COVID-19.^[24]^ At the same time, HCQ can be used as an oral hypoglycemic agent and has the effect of lowering blood glucose. However, there is no evidence-based medicine to confirm the effectiveness and safety of CQ and HCQ in the treatment of COVID-19 patients with diabetes. Therefore, we are trying to conduct a meta-analysis to provide high-quality evidence for CQ and HCQ treatment of COVID-19 patients with diabetes, and to inject new vitality into the clinical response to the COVID-19 epidemic.

### 3.1 Amendments

If the research process needs to be modified, we will update our protocol.

## Author contributions

The protocol was designed by YL under the guidance of CX and XF. All the authors participated in the study. The manuscript was drafted by YL and revised by XF and CX. All authors approved the final manuscript before submission.

**Conceptualization:** Yan Liu, Xiaoxu Fu and Chunguang Xie.

**Data curation:** Yan Liu, Chunguang Xie.

**Formal analysis:** Yan Liu, Xiaoxu Fu.

**Investigation:** Yan Liu, Xiaoxu Fu.

**Methodology:** Yan Liu, Xiaoxu Fu.

**Project administration:** Chunguang Xie.

**Software:** Yan Liu, Chunguang Xie.

**Visualization:** Yan Liu, Xiaoxu Fu.

**Writing – original draft:** Yan Liu.

**Writing – review & editing:** Chunguang Xie, Xiaoxu Fu.

## References

[1] Wu ZH Tang Y Cheng Q Diabetes increases the mortality of patients with COVID-19: a meta-analysis. Acta Diabetol 2020;1 6. 32583078 10.1007/s00592-020-01546-0 PMC7311595

[2] Bailes BK Diabetes mellitus and its chronic complications. AORN J 2002;76:266 86. 12194653 10.1016/s0001-2092(06)61065-x

[3] Lei Fang Karakiulakis George Roth Are patients with hypertension and diabetes mellitus at increased risk for COVID-19 infection? Lancet Respir Med 2020;8:e21. 32171062 10.1016/S2213-2600(20)30116-8 PMC7118626

[4] Chen N Zhou M Dong X Epidemiological and clinical characteristics of 99 cases of 2019 novel coronavirus pneumonia in Wuhan, China: a descriptive study. Lancet 2020;395:507 13. 32007143 10.1016/S0140-6736(20)30211-7 PMC7135076

[5] Wang D Hu B Hu C Clinical characteristics of 138 hospitalized patients with 2019 novel coronavirus-infected pneumonia in Wuhan, China. JAMA 2020;323:1061 9. 32031570 10.1001/jama.2020.1585 PMC7042881

[6] Huang C Wang Y Li X Clinical features of patients infected with 2019 novel coronavirus in Wuhan, China. Lancet 2020;395:497 506. 31986264 10.1016/S0140-6736(20)30183-5 PMC7159299

[7] Walls AC Park YJ Tortorici MA Structure, function, and antigenicity of the SARS-CoV-2 spike glycoprotein. Cell 2020;181:281 92. e6. 32155444 10.1016/j.cell.2020.02.058 PMC7102599

[8] Yang JK Lin SS Ji XJ Binding of SARS coronavirus to its receptor damages islets and causes acute diabetes. Acta Diabetol 2010;47:193 9. 19333547 10.1007/s00592-009-0109-4 PMC7088164

[9] Yang JK Feng Y Yuan MY Plasma glucose levels and diabetes are independent predictors for mortality and morbidity in patients with SARS. Diabet Med 2006;23:623 8. 16759303 10.1111/j.1464-5491.2006.01861.x

[10] Cortegiani A Ingoglia G Ippolito M A systematic review on the efficacy and safety of chloroquine for the treatment of COVID-19. J Crit Care 2020;57:279 83. 32173110 10.1016/j.jcrc.2020.03.005 PMC7270792

[11] Al-Bari MAA Targeting endosomal acidification by chloroquine analogs as a promising strategy for the treatment of emerging viral diseases. Pharmacol Res Perspect 2017;5:e00293 Published 2017 Jan 23. 28596841 10.1002/prp2.293 PMC5461643

[12] Lu H Drug treatment options for the 2019-new coronavirus (2019-nCoV). Biosci Trends 2020;14:69 71. 31996494 10.5582/bst.2020.01020

[13] Wang M Cao R Zhang L Remdesivir and chloroquine effectively inhibit the recently emerged novel coronavirus (2019-nCoV) in vitro. Cell Res 2020;30:269 71. 32020029 10.1038/s41422-020-0282-0 PMC7054408

[14] Colson P Rolain JM Raoult D Chloroquine for the 2019 novel coronavirus. Int J Antimicrob Agents 2020;55.3:105923. 32070753 10.1016/j.ijantimicag.2020.105923 PMC7134866

[15] Zhou N Pan T Zhang J Glycopeptide antibiotics potently inhibit cathepsin l in the late endosome/lysosome and block the entry of ebola virus, middle east respiratory syndrome coronavirus (MERS-CoV), and severe acute respiratory syndrome coronavirus (SARS-CoV). J Biol Chem 2016;291:9218 32. 26953343 10.1074/jbc.M116.716100 PMC4861487

[16] Singh AK Singh A Shaikh A Chloroquine and hydroxychloroquine in the treatment of COVID-19 with or without diabetes: a systematic search and a narrative review with a special reference to India and other developing countries. Diabetes Metab Syndr 2020;14:241 6. 32247211 10.1016/j.dsx.2020.03.011 PMC7102587

[17] Andreani J Le Bideau M Duflot I In vitro testing of combined hydroxychloroquine and azithromycin on SARS-CoV-2 shows synergistic effect. Microb Pathog 2020;145:104228. 32344177 10.1016/j.micpath.2020.104228 PMC7182748

[18] Moher D Shamseer L Clarke M Preferred reporting items for systematic review and meta-analysis protocols (PRISMA-P) 2015 statement. Syst Rev 2015;4:1. 25554246 10.1186/2046-4053-4-1 PMC4320440

[19] Riddle Matthew C American Diabetes Association standards of medical care in diabetes–2019. Diabetes Care 2019;42: Suppl 1: S34 60.30559230

[20] Higgins JPT Savović J Page MJ Chapter 8: Assessing risk of bias in a randomized trial. In: Higgins JPT, Thomas J, Chandler J, Cumpston M, Li T, Page MJ, Welch VA (editors). Cochrane Handbook for Systematic Reviews of Interventions version 6.0 (updated July 2019). Cochrane, 2019. Available from www.training.cochrane.org/handbook.

[21] Furlan AD Pennick V Bombardier C Editorial Board, Cochrane Back Review Group. 2009 updated method guidelines for systematic reviews in the Cochrane Back Review Group. Spine (Phila Pa 1976) 2009;34:1929 41. 19680101 10.1097/BRS.0b013e3181b1c99f

[22] Peters JL Sutton AJ Jones DR Contour-enhanced meta-analysis funnel plots help distinguish publication bias from other causes of asymmetry. J Clin Epidemiol 2008;61:991 6. 18538991 10.1016/j.jclinepi.2007.11.010

[23] Egger M Davey Smith G Schneider M Bias in meta-analysis detected by a simple, graphical test. BMJ 1997;315:629 34. 9310563 10.1136/bmj.315.7109.629 PMC2127453

[24] Gao J Tian Z Yang X Breakthrough: chloroquine phosphate has shown apparent efficacy in treatment of COVID-19 associated pneumonia in clinical studies. Biosci Trends 2020;14:72 3. 32074550 10.5582/bst.2020.01047

